# Anthropometric Failure and Associated Factors Among Infants Aged 6–8 Months in West Gojjam Zone, Ethiopia

**DOI:** 10.1002/fsn3.71548

**Published:** 2026-02-15

**Authors:** Shiferaw Birhanu, Getu Degu Alene, Yeshalem Mulugeta Demilew

**Affiliations:** ^1^ Department of Pediatrics and Child Health Nursing, School of Health Sciences, College of Medicine and Health Sciences Bahir Dar University Bahir Dar Ethiopia; ^2^ Department of Epidemiology and Biostatistics, School of Public Health, College of Medicine and Health Sciences Bahir Dar University Bahir Dar Ethiopia; ^3^ Department of Nutrition and Dietetics, School of Public Health, College of Medicine and Health Sciences Bahir Dar University Bahir Dar Ethiopia

**Keywords:** anthropometric failure, ethiopia, infants aged 6–8 months, West Gojjam

## Abstract

Childhood undernutrition in Ethiopia is a major public health concern. However, conventional indicators often miss concurrent growth failures, thereby underestimating the true burden among infants aged 6–8 months, a vulnerable group during the critical period of growth and complementary feeding. Therefore, this study aimed to assess undernutrition using the composite index of anthropometric failure and associated factors among infants aged 6–8 months in West Gojjam Zone, Northwest Ethiopia. A community‐based cross‐sectional study was conducted among 789 mothers with infants aged 6–8 months, selected using a cluster sampling technique in West Gojjam Zone, Northwest Ethiopia. Data were collected using a structured questionnaire, and anthropometric measurements were taken following standard procedures. Binary logistic regression analysis was performed to identify factors associated with undernutrition. Variables with a *p*‐value of < 0.05 were considered statistically significant. Nearly one‐third of infants aged 6–8 months were undernourished (29.2%; 95% CI: 26.0%, 32.5%) as measured by the composite index of anthropometric failure. In multivariable logistic regression, infants from households where the husband was the primary decision‐maker on resources [AOR = 2.36, 95% CI (1.51, 3.71)], infants being male [AOR = 4.05, 95% CI (2.87, 5.71)], being aged 7 months [AOR = 1.81, 95% CI (1.21, 2.70)], or 8 months [AOR = 1.86, 95% CI (1.23, 2.81)] compared with aged 6 months, having acute respiratory infection [AOR = 1.95, 95% CI (1.13, 3.39)], and low maternal self‐efficacy in complementary feeding [AOR = 1.51, 95% CI (1.05, 2.17)] were significant predictors of anthropometric failure. Therefore, interventions should promote shared household decision‐making, address illness‐related nutritional risks, and strengthen maternal confidence in complementary feeding.

**Trial Registration:** Registered at ClinicalTrials.gov (NCT05871346)

## Graphical abstract

This community‐based cross‐sectional study assessed undernutrition among 789 infants aged 6–8 months in West Gojjam Zone, Northwest Ethiopia, using composite index of anthropometric failure (CIAF). Nearly one‐third of infants experienced anthropometric failure, with stunting being the most common form. Male sex, older infant age, having acute respiratory infection (ARI), husband‐dominated household decision‐making, and low maternal self‐efficacy in complementary feeding were significant predictors of anthropometric failure.

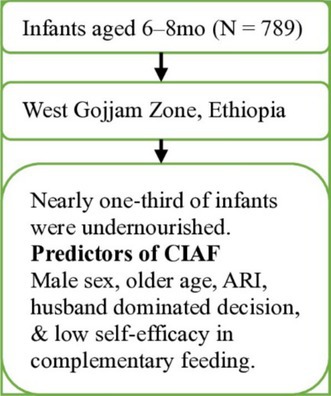

## 1 Introduction

Childhood undernutrition is a global public health burden that impairs children's physiological functioning, growth, and ability to respond to illness (Wells et al. 2019). Globally, its prevalence remains unacceptably high (Fanzo et al. 2018), with an estimated 149 million children under five being stunted and 45 million wasted in 2022 (World Health Organization). About 65% of stunted and 76% of wasted children live in lower‐middle‐income countries (UNICEF 2020; World Health Organization 2022). Furthermore, concurrent stunting and wasting affect 4.7% of children in low‐income countries and are associated with a 4.8‐fold increased risk of mortality (Victora et al. 2021).

In Sub‐Saharan Africa, undernutrition has increased over the past decades, with the number of stunted children increasing from 54.4 million in 2000 to 61.4 million in 2020 (Impact 2023). Ethiopia is one of the top‐ranked countries in the region for undernutrition (The Ethiopian Public Health Institute 2020) and faces a particularly high burden. According to the 2019 Ethiopian Mini Demographic Health Survey (EMDHS), 21%, 4.5%, and 16.2% of infants aged 6–8 months were stunted, wasted, and underweight, respectively (Rockville 2019). West Gojjam Zone is one of the most agriculturally productive zones in the Amhara region (Warner et al. 2019). Despite this, the area continues to face a high burden of child undernutrition (Amare et al. 2016; Kebede et al. 2021).

Millions of parents worldwide are struggling to provide nutritious and diverse foods (UNICEF 2024), in accordance with Sustainable Development Goal Target 2.2, which aims to “end all forms of child malnutrition by 2030” (Cepal 2016). The government of Ethiopia (2015) has also committed to end stunting among young children by 2030 through the “Seqota Declaration of Zero Hunger” (The Ethiopian Public Health Institute 2020). Although efforts have increased awareness of both the short‐ and long‐term consequences of undernutrition (Victora et al. 2021), substantial work is still required to meet the 2030 Targets.

The causes of undernutrition are multifactorial, with scientific evidence indicating that associated factors include a child's sex and age, parental education, morbidity, family size, child feeding practices, household resource decision‐making, food insecurity, and rural residence (Ayres et al. 2024; Fenta et al. 2021; Gebretsadik et al. 2024; Girma and Alenko 2020). Many studies in Ethiopia have assessed undernutrition using conventional indicators such as stunting, wasting, and underweight (Gagabo and Kuse 2023; Kuse and Debeko 2023; Raru et al. 2022; Sahiledengle et al. 2022; Woldekidan et al. 2024). However, these measures often overlook overlapping anthropometric failures and may underestimate the true burden (Gebretsadik et al. 2024). To address this limitation, the Composite Index of Anthropometric Failure (CIAF) was developed by Svedberg in 2000 and modified by Nandy et al. (2005) and Svedberg (2000). This index comprehensively assesses undernutrition by combining conventional indicators to provide an overall picture of growth deficits (Nandy and Svedberg 2012).

**FIGURE 1:**
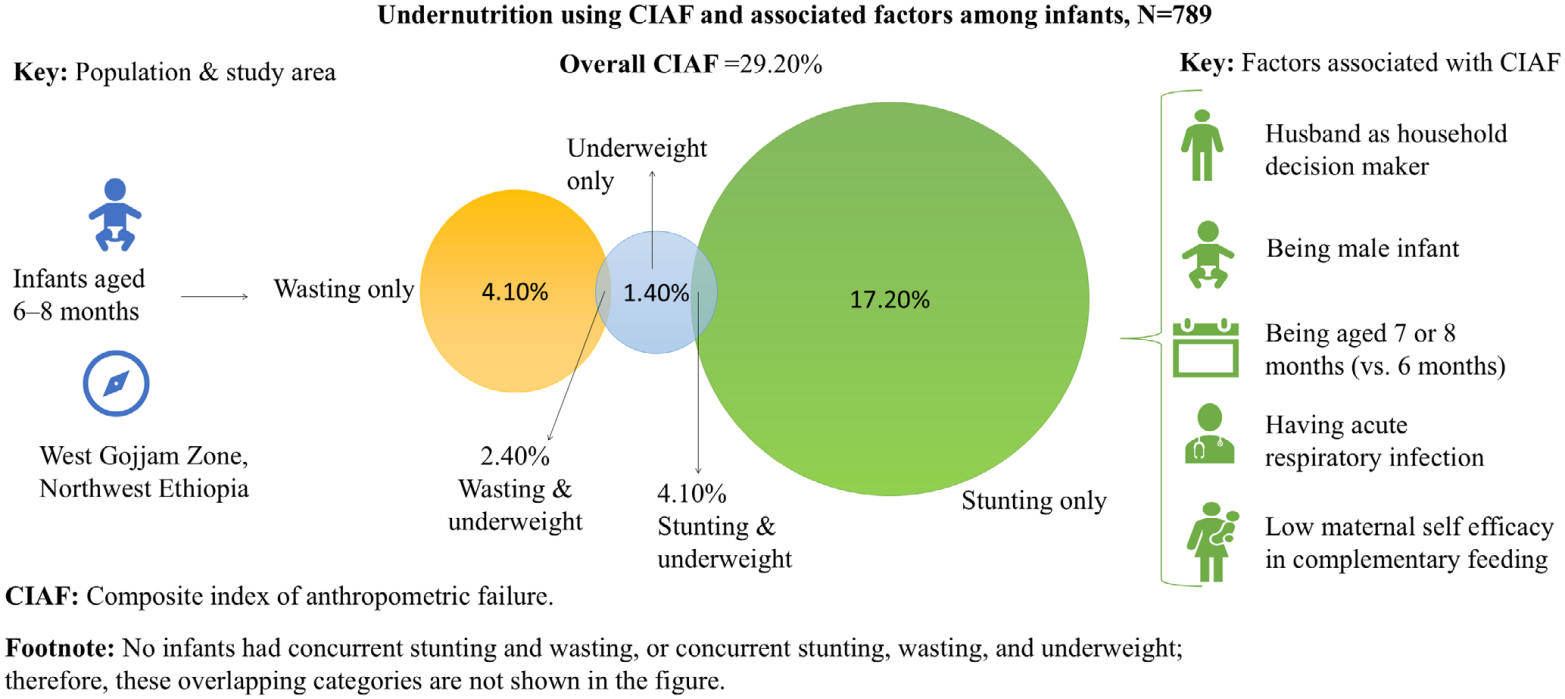
Graphical abstract illustrating undernutrition using the composite index of anthropometric failure (CIAF) and associated factors among infants aged 6–8 months in West Gojjam Zone, Northwest Ethiopia, 2023 (*N* = 789).

Despite its advantages, evidence on CIAF among infants aged 6–8 months in Ethiopia in general and in this study area in particular remains limited. This period is a critical transition, during which infants' susceptibility to undernutrition increases as breast milk alone no longer meets their nutritional needs, underscoring the need for age‐specific evidence to guide targeted interventions. Furthermore, there is limited research in Ethiopia on maternal psychological factors related to CIAF, such as perceived susceptibility, perceived severity, perceived benefits, perceived barriers, and self‐efficacy. This study therefore contributes to the literature by examining how these factors relate to the CIAF. It also serves as a baseline for the upcoming intervention titled “Effects of complementary feeding counseling on appropriate complementary feeding practices and child undernutrition in West Gojjam Zone, Northwest Ethiopia,” which is registered at ClinicalTrials.gov (NCT05871346). Hence, this study aimed to assess undernutrition using the composite index of anthropometric failure and to identify associated factors among infants aged 6–8 months in West Gojjam Zone, Northwest Ethiopia (Figure 1).

## 2 Materials and Methods

### 2.1 Study Design, Period, and Setting

A community‐based cross‐sectional study was conducted from June to December 2023 to determine CIAF prevalence rate and identify associated factors among infants aged 6–8 months in the rural areas of West Gojjam Zone, Amhara Region, Ethiopia. Finoteselam, the capital town of West Gojjam Zone, is located 389 km northwest of Addis Ababa, the capital city of Ethiopia. The zone comprises 22 districts, including 14 rural and 8 urban. As of 2023, the total population was estimated at 2,833,067, with 2,337,280 (82.5%) living in rural areas. Females made up 50.33% of the population, while infants accounted for 3.11%. There were 445 *kebeles* in the zone, of which 395 (88.8%) were rural. Approximately 72,690 infants resided in these rural *kebeles* (2,337,280 × 3.11%). The zone's healthcare infrastructure comprises 402 health posts, 108 health centers, and 8 public general hospitals.

### 2.2 Source and Study Population

The source population of this study included all mothers with infants aged 6–8 months in West Gojjam zone, while the study population comprised all mothers with infants aged 6–8 months from randomly selected *kebeles*.

### 2.3 Inclusion and Exclusion Criteria

As this study serves as a baseline for an upcoming intervention registered at ClinicalTrials.gov (NCT05871346), we included mothers with infants aged 6–8 months who had resided in the study area for at least 6 months prior to the survey, as local dietary practices are influenced by the cultural context. Mothers who planned to leave the study area during the trial period were excluded from the study.

### 2.4 Sample Size Determination

The sample size was calculated using both the single population proportion formula and Epi Info version 7 software, and the larger value was selected. For the single population proportion formula, we assumed a 95% confidence interval, 5% margin of error, 35.6% prevalence of underweight among infants aged 6–8 months from a previous study (Sewenet et al. 2022), a design effect of 2, and a 5% non‐response rate, resulting in a sample size of 741. Using Epi Info version 7, which assumed a 95% confidence interval, 80% power (1‐B), an unexposed to exposed ratio of 1:1, percentage outcome in the unexposed group of 20.3%, and an adjusted odds ratio of 1.93 for the predictor variables of underweight in the previous study (Sewenet et al. 2022), along with a design effect (DE) of 2 and a 5% non‐response rate, the calculated sample size was 802. Therefore, the final sample size for this study was taken as 802.

### 2.5 Sampling Procedure

Cluster sampling technique was used to select mothers with infants aged 6**–**8 months. From the 22 zonal districts, nine were excluded: one rural district (due to an ongoing nutrition intervention program) and eight urban districts (to maintain study homogeneity). From the remaining 13 rural districts, four (*Dembecha Zuria, Jabitehenan, Bure Zuria*, and *Yilmana Densa*) were randomly selected using simple random sampling (SRS). *Kebeles* were considered as clusters. We proportionally allocated and randomly selected 30 *kebeles* through SRS: 8 from *Dembecha Zuria*, 8 from *Jabitehenan*, 6 from *Bure Zuria*, and 8 from *Yilmana Densa*. Eligible infants in each *kebele* were screened through a house‐to‐house survey to confirm eligibility and their willingness to participate. All infants within selected clusters meeting the inclusion criteria were recruited using complete enumeration to minimize selection bias.

### 2.6 Data Collection Techniques and Instruments

Data were collected using a structured interviewer administered questionnaire adapted from the EMDHS 2019 (Rockville 2019), revised World Health Organization (WHO) infant and young child feeding (IYCF) practices indicators 2021 (World Health Organization 2021), and other similar studies (Das et al. 2022; Gebretsadik et al. 2024). The questionnaire included parental socio‐demographic and economic characteristics, child characteristics, feeding practices, history of infant morbidity, and maternal perceptions. To ensure participant comprehension, the English questionnaire was translated into Amharic (the local language). Eight trained health extension workers collected the data using face‐to‐face interviews in participants' homes. Written informed consent was obtained from mothers who could read and write. For those unable to do so, data collectors explained the purpose of the study in the local language. Following their agreement to participate, a fingerprint was obtained. Four master's‐level health professionals supervised the data collection process.

### 2.7 Variables

#### 2.7.1 Dependent Variable

Composite index of anthropometric failure (CIAF) was the dependent variable, coded as Failure = 1 and No failure = 0.

#### 2.7.2 Independent Variables

The independent variables included parental socio‐demographic and economic characteristics, child characteristics, feeding practices, history of infant morbidity, and maternal perceptions of child undernutrition and complementary feeding.

### 2.8 Operational Definitions

Variables related to complementary feeding practices, morbidity, and nutritional status were operationally defined in accordance with WHO recommendations. The operational definitions used in this study are summarized in Table 1.

**TABLE 1:** Operational definitions of key study variables.

Variable	Operational definition
Morbidity	Infant had ≥ 1 illness symptom (diarrhea, cough, or fever) within the 2 weeks preceding the survey; based on maternal report. No morbidity = negative responses for all symptoms (Rahman and Hossain 2022)
Timely introduction of complementary foods	Initiation of solid, semi‐solid, or soft foods (SSSFs) at 6–8 months of age. Introduction before 6 months is categorized as untimely (World Health Organization 2021)
Minimum dietary diversity (MDD)	Infant consumed foods from ≥ 5 of 8 WHO food groups in the previous 24 h; < 5 groups = does not meet MDD. Food groups: (1) breast milk; (2) grains/roots/tubers/plantains; (3) pulses, nuts, seeds; (4) dairy; (5) flesh foods; (6) eggs; (7) vitamin A–rich fruits/vegetables; (8) other fruits/vegetables (World Health Organization 2021)
Minimum meal frequency (MMF)	Breastfeeding infant consumed SSSFs at least twice in the previous day; < 2 times = unmet MMF (World Health Organization 2021)
Minimum acceptable diet (MAD)	Achieved when a breastfeeding infant meets both MDD and MMF; not achieved if either component is unmet MMF (World Health Organization 2021)
Egg and/or flesh food consumption	Infant consumed egg and/or flesh foods (meat, fish, poultry, organ meat) in the previous day; otherwise = not achieved (World Health Organization 2021)
Stunting	Length‐for‐age *z*‐score (LAZ) < −2 standard deviations (SD) according to WHO child growth standards. (World Health Organization 2006)
Wasting	Weight‐for‐length *z*‐score (WLZ) < −2 SD (World Health Organization 2006)
Underweight	Weight‐for‐age *z*‐score (WAZ) < −2 SD (World Health Organization 2006)

### 2.9 Measurements

#### 2.9.1 Anthropometric Measurements

The infant's length was measured in a recumbent position using the United Nations Children's Fund (UNICEF) portable wooden measuring board and recorded to the nearest 0.1 cm (Cashin and Oot 2018). Weight was measured using a UNICEF mechanical spring hanging scale with weighing pants and recorded to the nearest 0.01 kg (Cashin and Oot 2018).

#### 2.9.2 Composite Index of Anthropometric Failure (CIAF)

The composite index of anthropometric failure was used to assess overall infant undernutrition by combining the three conventional indicators: wasting, stunting, and underweight. Following the CIAF classification, infants were grouped into: (1) No failure (Group A), (2) Single failure (Group B: wasting only, Group F: stunting only, and Group Y: underweight only), (3) Double failure (Group C: wasting and underweight, and Group E: stunting and underweight), and (4) Triple failure (Group D: stunting, wasting, and underweight). Overall CIAF is the sum of single, double, and triple failures (Bose 2018; Nandy and Svedberg 2012) (Table 2).

**TABLE 2:** Classifications of the composite index of anthropometric failure (CIAF).

Groups	Failure type	LAZ	WLZ	WAZ	Stunting	Wasting	Underweight
A	No failure	Normal	Normal	Normal	No	No	No
B	Wasting only	Normal	< −2SD	Normal	No	Yes	No
C	Wasting and underweight	Normal	< −2 SD	< −2 SD	No	Yes	Yes
D	Stunting, wasting and underweight	< −2 SD	< −2 SD	< −2 SD	Yes	Yes	Yes
E	Stunting and underweight	< −2SD	Normal	< −2 SD	Yes	No	Yes
F	Stunting only	< −2SD	Normal	Normal	Yes	No	No
Y	Underweight only	Normal	Normal	< −2 SD	No	No	Yes
CIAF = B + C + D + E + F + Y							

Abbreviations: LAZ, Length‐for‐age *z*‐score; SD, Standard deviations; WAZ, Weight‐for‐age *z*‐score; WLZ, Weight‐for‐length *z*‐score.

*Source:* Adapted from Kutti (Bose 2018).

#### 2.9.3 Wealth Index

The household wealth index was assessed using principal components analysis (PCA) based on housing condition, source of drinking water, type of latrine, household assets, type of fuel used, livestock, and land ownership (Demilew et al. 2020; Mekonnen et al. 2022). Non‐dummy variables were classified into three categories, with the highest score coded as 1 and the two lower scores as 0. PCA assumptions were checked, and variables with a commonality value > 0.5 were used to generate factor scores (Demilew et al. 2020). Varimax rotation was applied to enhance interpretability by maximizing the variance explained by each component and clarifying the contribution of individual variables. Elven variables met this threshold and were retained for factor score calculation: radio, dining table, sofa or chairs, bed with cotton or sponge mattress, watch (by at least one household member), animal‐drawn cart (by at least one household member), flooring material of the house, cattle (cows or bulls), horses, donkeys, or mules, chickens, and proximity of drinking water to the home. The first principal component explained 19.0% of the variance, while the first five components collectively accounted for 65.6% of the total variance. These five components were retained to construct the wealth index, as they captured most of the household socioeconomic variation. Wealth scores were then categorized into tertiles to classify households as poor, medium, or rich.

#### 2.9.4 Psychological Factors

Maternal perceived susceptibility and severity of infant undernutrition, as well as perceived benefits, barriers, and self‐efficacy in complementary feeding were measured using composite items rated on a 5‐point Likert scale (1 = strongly disagree to 5 = strongly agree). Because the variables were ordinal and not normally distributed, a median was used as the cutoff point for categorization. For perceived susceptibility, severity, benefits, and self‐efficacy, scores above the median were classified as high, while scores at or below the median were classified as low.

Perceived barriers were treated differently, as lower scores represent a more favorable condition. Thus, mothers scoring at or below the median were categorized as having low (supportive) barriers, and those scoring above the median as having high (unsupportive) barriers. Assigning values equal to the cutoff to one of the two categories is an operational decision commonly used in epidemiologic studies to ensure consistency and interpretability (Altman and Royston 2006).

### 2.10 Data Quality Assurance

This study utilized a structured questionnaire adapted from standard data collection instruments. To ensure consistency, the instrument was first written in English, translated into Amharic, and then back‐translated into English. Experienced data collectors and supervisors were selected based on their prior expertise. They received training covering questionnaire content, study objectives, sampling techniques, and ethical issues. The tool was pretested and refined accordingly. Throughout the data collection, close supervision was maintained, including regular checks for data completeness. Data quality was maintained through careful recruitment, adequate training, and continuous supervision.

### 2.11 Data Processing and Analysis

Prior to statistical analysis, manual data coding and cleaning were performed to identify inconsistencies, missing values, and implausible anthropometric measurements. The data were entered into Epi‐Data software version 4.6 and then exported to SPSS 26 for analysis. Descriptive statistics such as frequency distribution and percentages were used to describe the study participants. To develop nutritional indicators, anthropometric data were converted into the WHO Anthro software, version 3.2.2.

Given the categorical nature of the outcome variable, a generalized linear mixed model (GLMM) was fitted to account for potential cluster‐level effects. The intercept‐only model revealed an intercept estimate of 0.213 and an intra‐cluster correlation coefficient (ICC) of 0.0608, suggesting minimal between‐cluster variation (only 6% of the total variance). This ICC, which is close to zero, indicates that 94% of the variation in CIAF was explained by individual‐level variables. When a level two variable (crop production at *kebele* level) was included, the random effect remained non‐significant (0.107, *p* = 0.313), confirming the limited influence of cluster‐level factors. Based on these findings, we determined that bivariable and multivariable logistic regression analyses were appropriate for identifying factors affecting CIAF.

Multicollinearity among predictor variables was assessed using variance inflation factors (VIF), which ranged from 1.006 to 2.220, indicating no significant multicollinearity among the explanatory variables. Model fitness was evaluated using the Hosmer‐Lemeshow goodness‐of‐fit test (*p* = 0.156). Variables with a *p*‐value of < 0.25 in the bivariable analysis were included in the multivariable analysis to assess their independent effects. Forward stepwise likelihood ratio method was employed during the multivariable logistic regression, and variables with a *p*‐value < 0.05 were considered statistically significant.

## 3 Results

### 3.1 Socio Demographic Characteristics

A total of 802 mothers with infants aged 6**–**8 months were enrolled, and data were collected from 789 participants (98.4% response rate); the mean maternal age was 29.66 ± 5.70 years. Over half of mothers (422, 53.5%) had no formal education. Nearly all mothers (786, 99.6%) were Orthodox Christian in religion, and 765 (97.0%) were married. Most mothers (632, 80.1%) reported making joint decisions with their husbands regarding household resources. Two‐thirds of households (522, 66.2%) had fewer than six residents. Just over half of the infants (401, 50.8%) were female. In half of the households (398, 50.4%), there were two children under five. Additionally, 268 participants (34.0%) were from households classified as medium socioeconomic status (Table 3).

**TABLE 3:** Socio‐demographic characteristics of mothers with infants aged 6**–**8 months in West Gojjam Zone, Northwest Ethiopia, 2023 (*N* = 789).

Variables	Category	Frequency	Percent
Mother's age in year	≤ 24	148	18.8
	25–34	454	57.5
	≥ 35	187	23.7
Religion	Orthodox	786	99.6
	Others	3	0.4
Mather's education	No formal education	422	53.5
	Primary education	289	36.6
	Secondary/higher	78	9.9
Current marital status	Married	765	97.0
	Others^a^	24	3.0
Husband's education (765)	No formal education	235	30.7
	Primary education	470	61.5
	Secondary/higher	60	7.8
Decision‐making on household resources	Husband	112	14.2
	Wife	45	5.7
	Jointly	632	80.1
Family size	< 6	522	66.2
	≥ 6	267	33.8
Sex of infant	Male	388	49.2
	Female	401	50.8
Age of infant in months	6 months	288	36.5
	7 months	275	34.9
	8 months	226	28.6
Number of children under five	One	365	46.3
	Two	398	50.4
	Three	26	3.3
Wealth index	Poor	258	32.7
	Medium	268	34.0
	Rich	263	33.3

^a^ Single, divorced, widowed.

### 3.2 Infant Feeding Status and Health‐Related Characteristics

Over three‐fourths of infants (632, 80.1%) were introduced to SSSFs at the recommended age of 6–8 months. Two‐thirds of infants (529, 67.0%) were fed two or more meals during the previous day. However, only 107 (13.6%) and 104 (13.2%) of infants met the MDD and MAD, respectively, and 188 (23.8%) of infants had consumed eggs and/or flesh foods during the previous day. Most infants (658, 83.4%) received age‐appropriate vaccines (Table 4).

**TABLE 4:** Feeding status and health‐related characteristics among infants aged 6**–**8 months in West Gojjam Zone, Northwest Ethiopia, 2023 (*N* = 789).

Variables	Category	Frequency	Percent
SSSFs introduced at 6–8 months	Yes	632	80.1
	No	157	19.9
Minimum dietary diversity	Met	107	13.6
	Not met	682	86.4
Minimum meal frequency	Met	529	67.0
	Not met	260	33.0
Egg and/or flesh food consumption	Yes	188	23.8
	No	601	76.2
Minimum acceptable diet	Yes	104	13.2
	No	685	86.8
Bottle feeding	Yes	87	11.0
	No	702	89.0
Diarrhea within 2 weeks preceding the survey	Yes	106	13.4
	No	683	86.6
Fever within 2 weeks preceding the survey	Yes	70	8.9
	No	719	91.1
ARI within 2 weeks preceding the survey	Yes	67	8.5
	No	722	91.5
Infant received age‐appropriate vaccines	Yes	658	83.4
	No	131	16.6

Abbreviations: ARI: Acute respiratory infection, SSSFs: Solid, semi‐solid, or soft foods.

### 3.3 Prevalence and Distribution of Undernutrition Using CIAF

**FIGURE 2:**
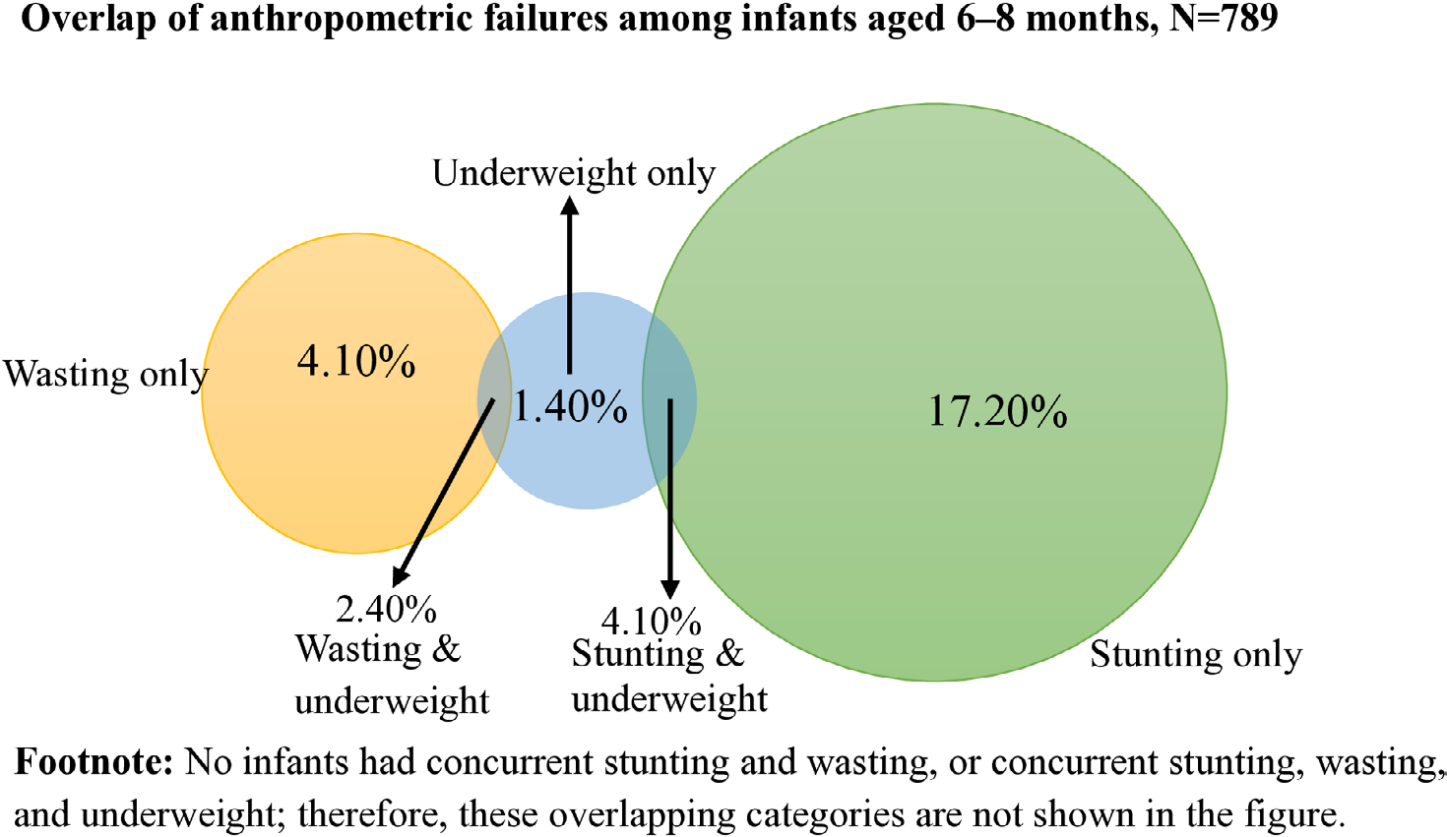
Overlap of anthropometric failures among infants aged 6–8 months in West Gojjam Zone, Northwest Ethiopia, 2023 (*N* = 789).

The overall CIAF prevalence rate among infants aged 6**–**8 months was 29.2%. Single anthropometric failures were most common (22.7%), particularly stunting only (17.2%), followed by wasting only (4.1%) and underweight only (1.4%). Double failures were less frequent (6.5%), occurring mainly as stunting and underweight (4.1%) and wasting with underweight (2.4%). No cases of triple failure were observed, and no infants had concurrent stunting and wasting. Stunting was the most prevalent form of failure across all age groups. Most anthropometric failures occurred more often among males. Overall, the CIAF was substantially higher among males (42.2%) than females (16.5%). Detailed distributions are presented in Table 5, while the overlap of failures is illustrated in Figure 2.

**TABLE 5:** Prevalence of the composite index of anthropometric failure by age and sex among infants aged 6**–**8 months in West Gojjam Zone, Northwest Ethiopia 2023 (*N* = 789).

Categories of CIAF	Infant's age in month			Sex		
	6	7	8	Male	Female	Total
No failure (A)	222 (77.1)	189 (68.7)	148 (65.5)	224 (57.8)	335 (83.5)	559 (70.8)
Wasting only (B)	10 (3.5)	11 (4.0)	11 (4.9)	16 (4.1)	16 (4.0)	32 (4.1)
Wasting and underweight (C)	2 (0.7)	7 (2.6)	10 (4.4)	18 (4.6)	1 (0.3)	19 (2.4)
Stunting, wasting and underweight (D)	—	—	—	—	—	—
Stunting and underweight (E)	8 (2.8)	13 (4.7)	11 (4.9)	28 (7.2)	4 (1.0)	32 (4.1)
Stunting only (F)	45 (15.6)	47 (17.1)	44 (19.5)	93 (24.0)	43 (10.7)	136 (17.2)
Underweight only (Y)	1 (0.3)	8 (2.9)	2 (0.8)	9 (2.3)	2 (0.5)	11 (1.4)
CIAF = B + C + D + E + F + Y	66 (22.9)	86 (31.3)	78 (34.5)	164 (42.2)	66 (16.5)	230 (29.2)

Abbreviation: CIAF, Composite index of anthropometric failure.

### 3.4 Factors Associated With CIAF

Variables with a *p*‐value < 0.25 in the bivariable analysis were included in the multivariable model. These included: decision‐making on household resources, family size, infant sex and age, respiratory infection (ARI) within 2 weeks preceding the survey, initiation of SSSFs at 6**–**8 months, minimum meal frequency, wealth index, maternal perceived severity of child undernutrition, perceived benefits of complementary feeding, and self‐efficacy in complementary feeding.

In multivariable analysis, decision‐making on household resources, infant sex and age, ARI, and maternal perceived self‐efficacy in complementary feeding were the independent predictors of CIAF. Infants from households where the husband was the primary decision‐maker on resources had 2.4 times [AOR = 2.36, 95% CI: (1.51, 3.71)] higher odds of experiencing CIAF compared with those from households with joint decision‐making. Male infants were 4 times [AOR = 4.05, 95% CI: (2.87, 5.71)] more likely to have CIAF than female infants. Infants aged 7 or 8 months were 1.8 times [AOR = 1.81, 95% CI: (1.21, 2.70)] and 1.9 times [AOR = 1.86, 95% CI: (1.23, 2.81)] more likely to experience CIAF, respectively, than infants aged 6 months. Infants who had ARI within 2 weeks before the survey were 2 times [AOR = 1.95, 95% CI: (1.13, 3.39)] more likely to experience CIAF than their counterparts. Infants whose mothers had low self‐efficacy in complementary feeding were 1.5 times [AOR = 1.51, 95% CI: (1.05, 2.17)] more likely to experience CIAF than their counterparts (Table 6).

**TABLE 6:** Factors associated with the CIAF among infants aged 6**–**8 months in West Gojjam Zone, Northwest Ethiopia, 2023 (*N* = 789).

Variables	CIAF		COR (95% CI)	AOR (95% CI)
	Failure *n* (%)	No failure *n* (%)		
Decision‐making				
Husband	46 (41.1)	66 (58.9)	1.835 (1.211, 2.778)*	2.361 (1.505, 3.706)**
Wife	10 (22.2)	35 (77.8)	0.752 (0.365, 1.551)	0.745 (0.346, 1.605)
Jointly	174 (27.5)	458 (72.5)	1	1
Family size				
≤ 5 people	161 (30.8)	361 (69.2)	1	
≥ 6 people	69 (25.8)	198 (74.2)	0.781 (0.561, 1.088)	
Sex of infant				
Male	164 (42.3)	224 (57.7)	3.716 (2.666, 5.180)**	4.046 (2.866, 5.709)**
Female	66 (19.7)	335 (80.3)	1	1
Infant's age in month				
6 months	66 (22.9)	222 (77.1)	1	1
7 months	86 (31.3)	189 (68.7)	1.531 (1.052, 2.227)*	1.811 (1.214, 2.702)*
8 months	78 (34.5)	148 (65.5)	1.773 (1.202, 2.614)*	1.857 (1.226, 2.810)*
ARI within 2 weeks				
Yes	27 (40.3)	40 (59.7)	1.726 (1.032, 2.887)*	1.952 (1.125, 3.386)*
No	203 (28.1)	519 (71.9)	1	1
SSSFs at 6–8 months				
No	37 (23.6)	120 (76.4)	0.701 (0.468, 1.052)	
Yes	193 (30.5)	439 (69.5)	1	
Minimum meal frequency				
Not met	65 (25.0)	195 (75.0)	0.735 (0.526, 1.029)	
Met	165 (31.2)	364 (68.8)	1	
Wealth index				
Poor	71 (27.5)	187 (72.5)	0.730 (0.502, 1.060)	
Medium	69 (25.7)	199 (74.3)	0.666 (0.459, 0.969)*	
Rich	90 (34.2)	173 (65.8)	1	
Perceived susceptibility to child undernutrition				
Low	165 (29.2)	400 (70.8)	1.009 (0.718, 1.419)	
High	65 (29.0)	159 (71.0)	1	
Perceived severity of child undernutrition				
Low	165 (31.1)	366 (68.9)	1.339 (0.957, 1.873)	
High	65 (25.2)	193 (74.8)	1	
Perceived benefits of complementary feeding				
Low	152 (30.9)	340 (69.1)	1.255 (0.910, 1.731)	
High	78 (26.3)	219 (73.7)	1	
Perceived barriers to complementary feeding				
Low	114 (27.7)	298 (72.3)	1	
High	116 (30.8)	261 (69.2)	1.162 (0.854, 1.580)	
Perceived self‐efficacy in complementary feeding				
Low	168 (31.1)	372 (68.9)	1.362 (0.969, 1.914)	1.509 (1.050, 2.170)*
High	62 (24.9)	187 (75.1)	1	1

*Note:* Variables without AOR values did not meet the *p* < 0.25 criterion for inclusion in the multivariable model.

Abbreviations: AOR, Adjusted odds ratio; ARI, Acute respiratory infection; CI, Confidence interval; CIAF, Composite index of anthropometric failure; COR, Crude odds ratio; SSSFs, Solid, semi‐solid, or soft foods.

* *p* < 0.05.

** *p* < 0.001.

## 4 Discussion

Conventional anthropometric indicators provide an incomplete understanding of child undernutrition, as a single child may experience several forms of nutritional failure simultaneously. Therefore, this study assessed undernutrition using the composite index of anthropometric failure and examined its associated factors among infants aged 6**–**8 months in rural areas of West Gojjam Zone, Amhara region, Northwest Ethiopia.

The overall prevalence rate of CIAF in this age group was 29.2%. One possible explanation is that breast milk alone meets only about 60% of infants' nutritional requirements at this age (World Health Organization 2021). Without adequate complementary feeding, infants become vulnerable to anthropometric failure. Evidence showed that undernutrition rises sharply after 6 months of age when complementary foods are not introduced to meet the remaining nutrient gap (World Health Organization 2003). The prevalence rate observed in our study is comparable to the 29.6% reported in Indonesia among children aged 0–24 months (Permatasari and Chadirin 2022). However, it is lower than findings from Southwest Ethiopia (57.3% among children aged 6**–**23 months) (Gebretsadik et al. 2024), 40.7% in analysis of the 2019 EMDHS (Ayres et al. 2024), and 52.2% among children under five in Pakistan (Balogun et al. 2021). The higher prevalence figures may partly reflect differences in the age groups studied, as the risk of anthropometric failure tends to increase with age possibly due to growing nutritional demands and developmental changes.

Infants living in households with husband‐dominated resource decision‐making were more likely to experience CIAF compared to those in households where decisions were made jointly. Limited involvement of mothers in household decisions may reduce resource allocation for complementary feeding. This finding aligns with studies from Southwest Ethiopia showing increased undernutrition in households where mothers lack decision‐making power (Girma and Alenko 2020) and with EDHS 2011 analyses reporting higher odds of stunting among children in male‐headed households (Haile et al. 2016).

Male infants had higher odds of CIAF than female infants, consistent with multiple studies reporting greater anthropometric failure among males (Ahmadi et al. 2018; Ayres et al. 2024; Fenta et al. 2021; Gebretsadik et al. 2024; Haile et al. 2016). This pattern may be explained by the higher proportion of male preterm births (Cooperstock and Campbell 1996; Dewey and Brown 2003), as well as greater biological vulnerability among male infants to infection, inflammation, and neonatal complications, which can impede growth. Recent evidence also suggests that male infants experience higher morbidity and are more sensitive to inflammatory insults and early brain injury (Kelly et al. 2023; Thompson et al. 2024). However, contrasting evidence from India reported no significant sex difference in undernutrition (Khan and Raza 2016).

The likelihood of CIAF increased with age, with infants aged 7 or 8 months being more affected than those aged 6 months. This pattern may reflect increased nutritional requirements during periods of rapid growth (World Health Organization 2021) and possible delays in the timely introduction of complementary foods to bridge the nutrient gap left by breastfeeding alone. These findings are consistent with analyses of EDHS 2011 and 2014 data and the 2019 EMDHS (Ayres et al. 2024; Endris et al. 2017; Haile et al. 2016), as well as a study from India demonstrating a significant association between increasing age and undernutrition (Khan and Raza 2016).

Infants who had acute respiratory infection within 2 weeks preceding the survey were more likely to experience CIAF than those without such infection. This is consistent with the vicious cycle between undernutrition and infection: infections deplete essential nutrients needed for immune function, while undernutrition increases susceptibility to recurrent infections, exacerbates illness severity, and delays recovery (Chiabi et al. 2018; Walson and Berkley 2018). Similar findings have been reported from Ethiopia, Burkina Faso, and Indonesia (Bidira et al. 2021; Faridasari et al. 2020; Poda et al. 2017). Illness can also reduce appetite and food intake while increasing metabolic demands due to fever and labored breathing, leading to negative energy balance and loss of nutrient stores. This is supported by a study among rural Kenyan children with lower respiratory tract infections (Neumann et al. 2012).

Maternal self‐efficacy in complementary feeding was another important predictor of CIAF. Mothers with low self‐efficacy were more likely to have infants experiencing anthropometric failure. Consistently, studies in Indonesia have shown that low maternal self‐efficacy is associated with suboptimal nutritional parenting patterns and inadequate child feeding, whereas higher maternal self‐efficacy is linked to providing sufficient nutrition and better child nutritional status (Khasanah et al. 2024; Ratnaningsih et al. 2018). This may occur because low self‐efficacy can undermine a mother's confidence in performing recommended feeding behaviors (Champion and Skinner 2008).

This study demonstrates that husband‐dominated resource decision‐making, low maternal self‐efficacy in complementary feeding, and recent ARI are associated with CIAF. These results underscore the need for multisectoral interventions addressing structural and behavioral determinants of child nutrition. Evidence from Burkina Faso suggests that empowering women can reduce child wasting (Heckert et al. 2019), while studies from Ethiopia and South Africa similarly show that maternal involvement in household decision‐making is associated with improved child nutritional outcomes (Adediran 2024; Mekonnen et al. 2021). In addition, behavior‐change communication interventions targeting complementary feeding have been shown to enhance feeding practices among young children (Gebretsadik et al. 2025). Integrated programs that combine women's empowerment, improved complementary‐feeding practices, and infection‐prevention strategies may therefore help reduce anthropometric failure. Future studies should evaluate the effect of such combined interventions for reducing CIAF in rural Ethiopia and similar contexts.

This study provides a focused assessment of CIAF among infants aged 6**–**8 months using a representative sample. It also examined maternal psychological factors and found that maternal self‐efficacy in complementary feeding was an important predictor, demonstrating an association with CIAF. However, certain limitations should be considered. Dietary assessment relied on maternal 24‐h recall, which may be subject to recall and social‐desirability bias. The exclusive focus on rural populations limits generalizability to urban settings. Additionally, due to limited evidence specific to this age group, some comparisons were made with studies covering broader age ranges (6–23 months or under 59 months). These factors should be considered when interpreting the results.

## 5 Conclusion

In this study, nearly one‐third of infants aged 6**–**8 months experienced anthropometric failure as measured by the composite index of anthropometric failure (CIAF). The CIAF combines conventional indicators to provide a more comprehensive assessment of growth failure in this age group. Factors independently associated with CIAF were husband‐dominated resource decision‐making, male sex, older infant age (7–8 months vs. 6 months), recent acute respiratory infection, and low maternal self‐efficacy in complementary feeding. These findings underscore the need for interventions that promote shared household decision‐making, address nutrition‐related risks of illness, and strengthen maternal confidence in complementary feeding.

## Author Contributions

Shiferaw Birhanu, Getu Degu Alene, and Yeshalem Mulugeta Demilew made substantial contributions to the conception, data acquisition, analysis, and investigation for the manuscript. Shiferaw Birhanu drafted the manuscript. All authors critically revised the manuscript for important intellectual content and approved the final version for submission and publication. All authors agreed to be accountable for all aspects of the work, ensuring that questions related to the accuracy or integrity of any part of the work are appropriately investigated and resolved.

## Funding

The authors have nothing to report.

## Ethics Statement

This study was approved by the Bahir Dar University, College of Medicine and Health Sciences Institutional Review Board (IRB). A letter of support was obtained from the Amhara Public Health Institute. Relevant officials were also informed hierarchically, and their permission letters were obtained.

## Consent

Written informed consent was obtained from literate mothers. For mothers who could not read and write, voluntary participation was ensured by providing a detailed explanation of the study's purpose in a language they understood. Following their agreement to participate, a fingerprint was obtained. Confidentiality was maintained by excluding personal identifiers from the data collection questionnaire, and data were kept in a locked board.

## Conflicts of Interest

The authors declare no conflicts of interest.

## Data Availability

Upon request, the corresponding author will provide access to the datasets used in this study.
